# A first Japanese case of neuroendocrine prostate cancer accompanied by lung and brain metastasis with somatic and germline BRCA2 mutation

**DOI:** 10.1111/pin.12860

**Published:** 2019-10-20

**Authors:** Takeo Kosaka, Hiroshi Hongo, Eriko Aimono, Kazuhiro Matsumoto, Tetsu Hayashida, Shuji Mikami, Hiroshi Nishihara, Mototsugu Oya

**Affiliations:** ^1^ Department of Urology Keio University School of Medicine Tokyo Japan; ^2^ Genomics Unit, Keio Cancer Center Keio University School of Medicine Tokyo Japan; ^3^ Department of Surgery Keio University School of Medicine Tokyo Japan; ^4^ Division of Diagnostic Pathology Keio University Hospital Tokyo Japan

**Keywords:** BRCA2, DNA damage repair genes, NEPC, platinum‐based chemotherapy

## Abstract

Germline mutations and copy number changes in DNA damage repair (DDR) genes such as *BRCA2* are associated with aggressive forms of prostate cancer (PCa). Although the prevalence of BRCA2 variants in PCa is increasing in Japan, the genomic and biological implications in Japanese patients are unclear. An 81‐year‐old male presented with prostatic adenocarcinoma with neuroendocrine differentiation accompanied by metastatic lung nodule and brain metastases. Platinum‐based doublet chemotherapy combined with etoposide resulted in partial and complete remissions of brain and lung metastases, respectively. Next‐generation sequencing of biopsy and peripheral blood samples demonstrated a somatic BRCA2 mutation at c.7008‐2A>C and a germline mutation at p.E2877*. The patient's son had been diagnosed with breast cancer 2.5 years ago and was found to have the same germline BRCA2 mutation. BRCA2 mutation increases the risks of aggressive PCa and other cancer types in Japanese males. These forms may be highly responsive to platinum‐based chemotherapy.

**Abbreviations**
DDR: DNA damage repair
HR: homologous recombination
DSBs: double strand breaks
NEPC: adenocarcinoma of the prostate with neuroendocrine differentiation
PCa: prostate cancer

## 1 INTRODUCTION

Genome integrity is maintained by numerous DNA damage repair genes (DDRs), and germline mutations in DDRs greatly increase the risks of several cancer types, including prostate, breast, and ovarian cancer.^1^ Accumulating evidence also suggests that germline mutations and copy number changes in DDR‐related genes such as BRCA1, BRCA2, ATM, CHEK2, and FANCA increase the risk for aggressive forms of prostate cancer (PCa).^1^ Moreover, somatic events caused by DDRs mutations are associated with aggressive PCa phenotypes.^2^, ^3^ However, these associations were established in Western patient cohorts, whereas the genomic and biological implications in Japanese patients are unclear, despite the growing prevalence of PCa in Japan.

We present the case of an 81‐year‐old male with metastatic adenocarcinoma of the prostate with neuroendocrine differentiation (NEPC) accompanied by brain metastasis and a *BRCA2* mutation. Moreover, he had a 51‐year‐old son with breast cancer history sharing the same mutation, implicating *BRCA2* mutations in elevated cancer risk among Japanese males.

## 2 CASE PRESENTATION

**Figure 1:**
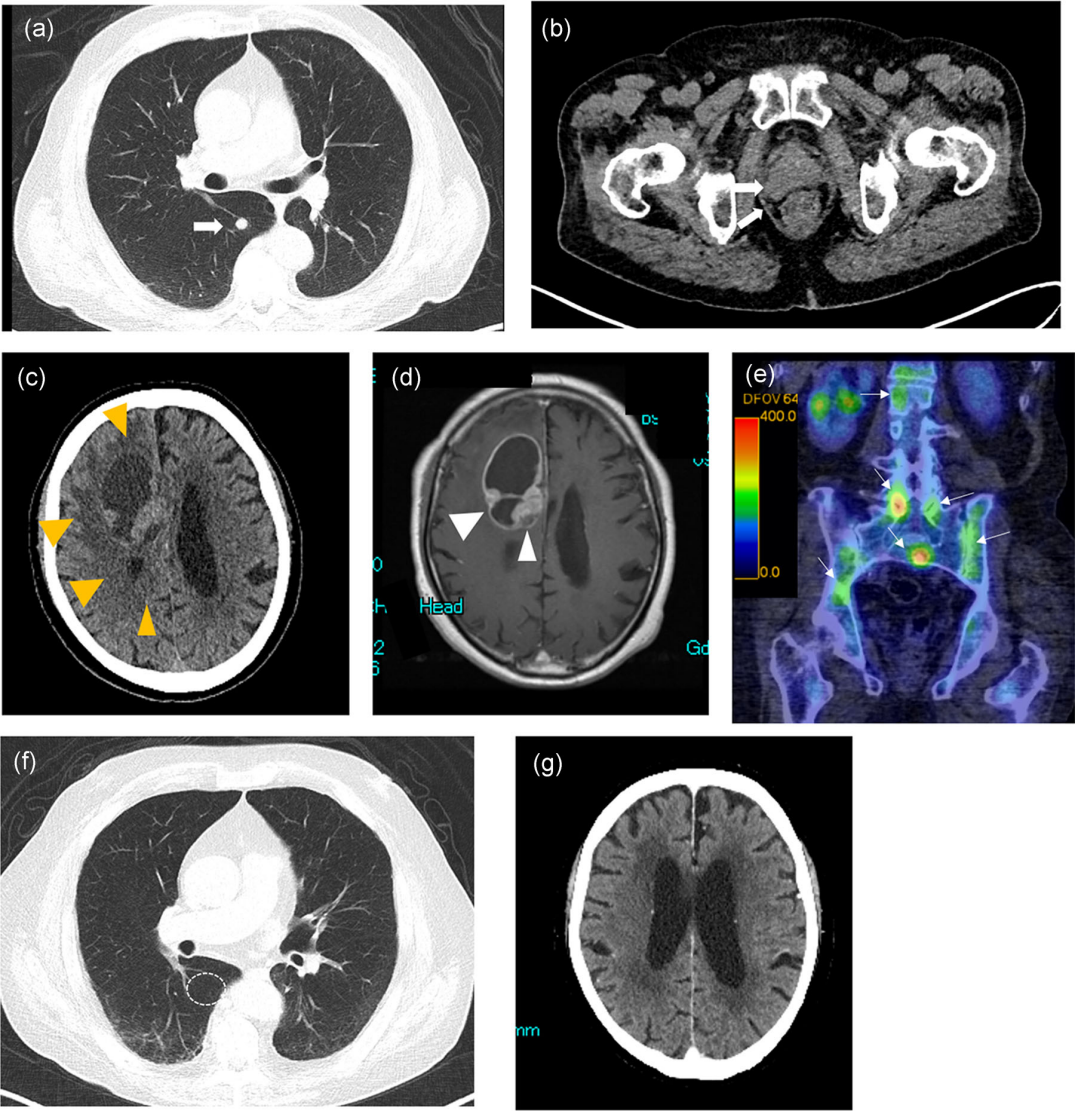
Diagnostic imaging findings on presentation. (**a**) PET image showing metastatic lung nodules in the right lobe. (**b**) CT image of the prostate showing the irregular shape. (**c,d**) Brain CT (**c**) and MRI (**d**) images showing signs of frontal lobe metastasis. (**e**) 99mTc‐HMDP bone scintigraphy revealing multiple metastases in the spinal bone. (**e,f**) Imaging results after treatment. Chest (**e**) and brain (**f**) CT after platinum‐based doublet chemotherapy combined with etoposide. CT shows partial remission of brain metastasis and complete remission of pulmonary metastasis. CT, computed tomography; MRI, magnetic resonance imaging; PET, positron emission tomography.

An 81‐year‐old male presented with signs of possible lung cancer on positron emission tomography (PET) scans for follow‐up monitoring of chronic respiratory obstructive disease (Fig. 1a). A computed tomography (CT) scan also revealed an irregularly shaped prostatic grand (Fig. 1b), so he was referred to our urology department for detailed examination. Although serum prostate‐specific antigen (PSA) concentration was only 0.78 ng/mL, digital rectal examination revealed a stony hard nodule in the right lobe, suggesting PCa. On admission to the hospital for prostate biopsy, he was found to have reduced motivation. Therefore, emergent head CT and magnetic resonance imaging (MRI) were conducted that also identified an irregular ring‐like enhancing mass in the right frontal robe (Fig. 1c and d), suggesting metastatic brain cancer. Thus, emergent cerebral decompression and craniotomy biopsy were performed by a neurosurgeon, and prostate needle biopsy was performed at the same time. Bone scan also identified multiple bone metastases (Fig. 1e).

## 3 PATHOLOGICAL FINDINGS

**Figure 2:**
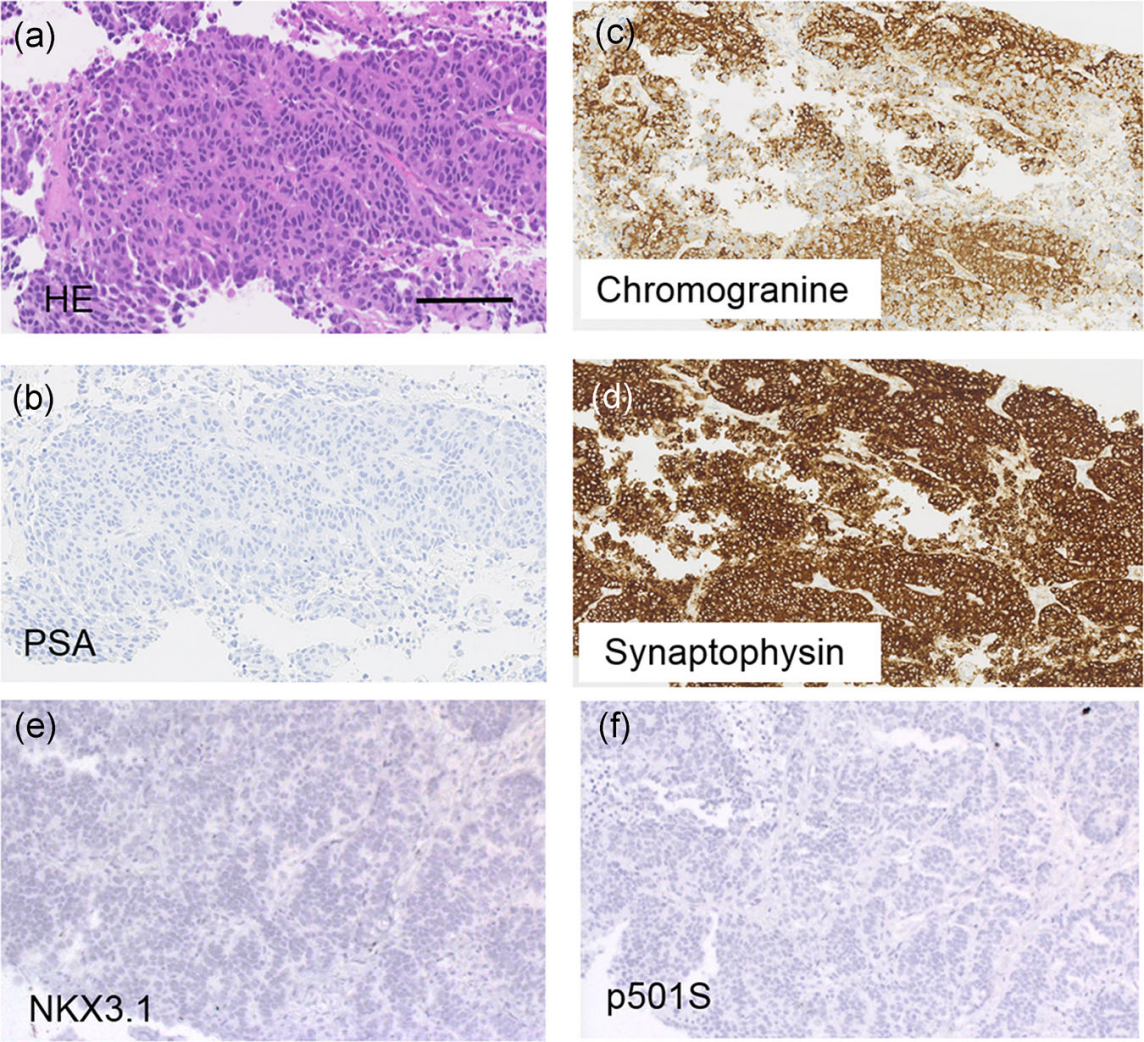
Histology and immunohistochemistry of the prostate biopsy specimen. (**a**) Representative images of hematoxylin and eosin (HE) staining. (**b–f**) Immunohistochemistry for PSA (**b**), chromogranine (**c**), synaptophysin (**d**), NKX3.1 (**e**), and p501S (**f**). Bars indicate 100 μm. PSA, prostate‐specific antigen.

**Figure 3:**
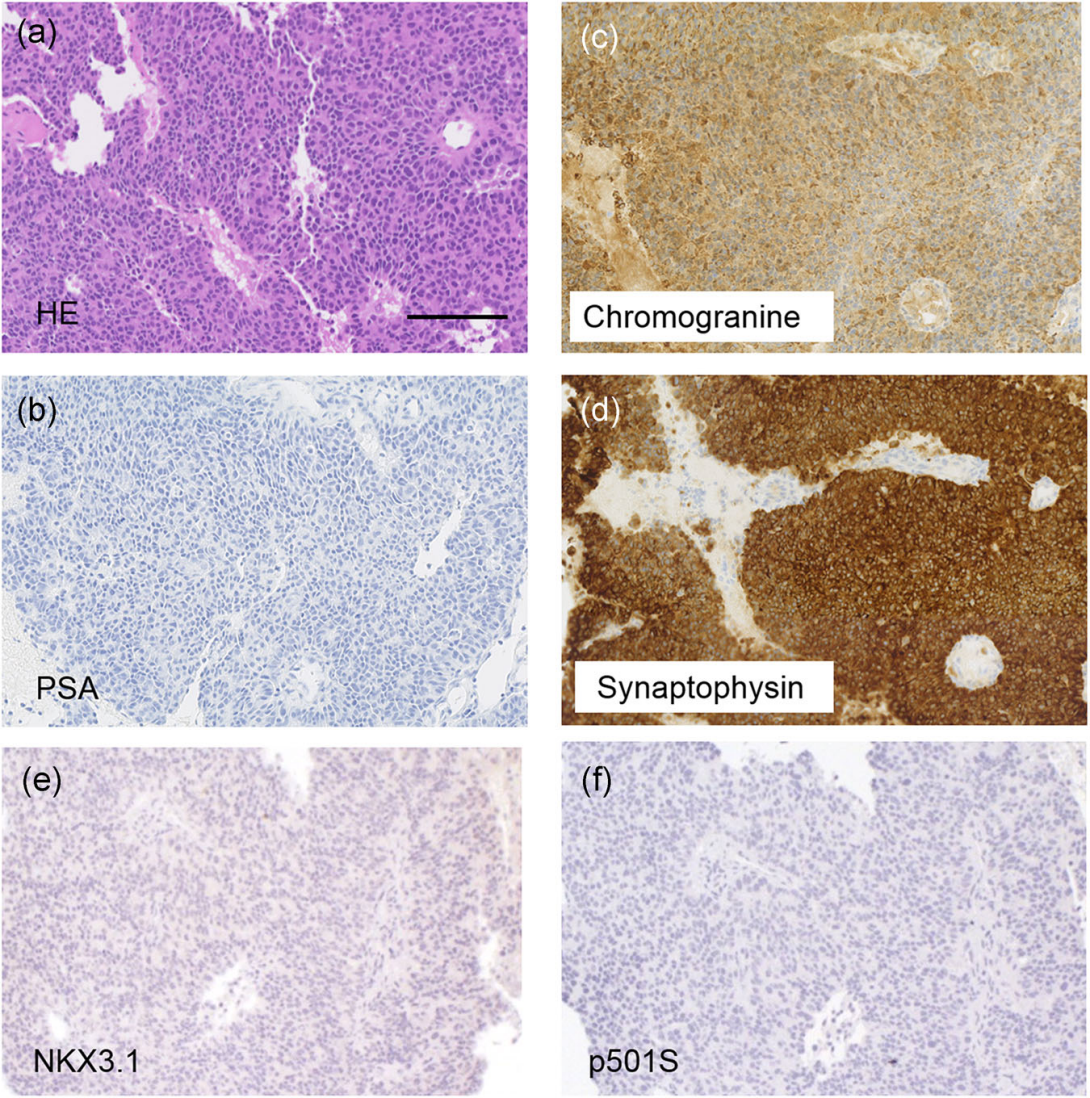
Histology and immunohistochemistry of the brain biopsy specimen. (**a**) Representative images of hematoxylin and eosin (HE) staining. (**b–d**) Immunohistochemical staining for PSA (**b**), chromogranine (**c**), and synaptophysin (**d**), NKX3.1 (**e**), and p501S (**f**). Bars indicate 100 μm. PSA, prostate‐specific antigen.

Pathological examination revealed that cells in prostatic adenocarcinoma exhibited solid and trabecular patterns with a partial glandular structure; thus, the patient was diagnosed with poorly differentiated adenocarcinoma, with a Gleason grade of 9 (4 + 5). According to an immunohistochemical analysis, he tested negative for PSA, NKX3.1, and p501s, the common markers of prostatic adenocarcinoma, but was tested positive for synaptophysin and chromogranin A (Fig. 2a–f). Three pathologists reached a consensus regarding the final pathological diagnosis of metastatic adenocarcinoma of NEPC. The metastatic brain tumor and prostatic adenocarcinoma had similar histological (appearance of solid and trabecular patterns) and immunohistochemical characteristics (Fig. 3a–f); therefore, the pathologist established a final diagnosis of metastatic adenocarcinoma of NEPC (Fig. 3a–f).^4^ Based on the results, the clinical stage was identified to be cT3bN1M1c.

The patient received androgen deprivation and platinum‐based doublet chemotherapy combined with etoposide. Follow‐up CT after five cycles of chemotherapy (delivered over 5 months) revealed partial remission of brain metastasis and complete remission of pulmonary metastasis (Fig. 1f). After the sixth cycle, PSA was less than 0.01 ng/mL. Serum neuron‐specific enolase, a biomarker for NEPC, was also reduced to less than 11.8 ng/mL at 5 months.

**Figure 4:**
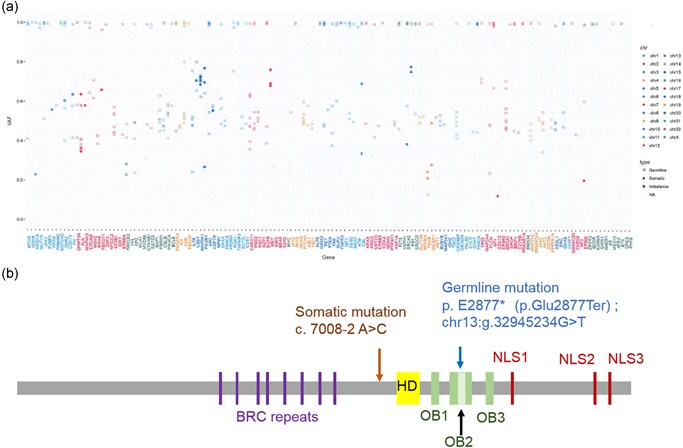
Genomic profiling of cancer‐related genes (**a, b**). The horizontal axis indicates the examined genes, and the vertical axis indicates the variant allele frequency (**a**). BRCA2 mutation sites. Profiling revealed a somatic mutation at c. c.7008‐2A>C and a germline mutation at p.E2877*(p.Glu2877Ter). The patient's son shared the germline mutation (**b**). Germline mutation.

Cancer‐related gene profiling of the formalin‐fixed paraffin‐embedded prostate tumor specimen and peripheral blood (as a control) was performed using next‐generation sequencing as previously reported^5^, ^6^ (Fig. 4a). We identified a somatic *BRCA2* mutation at c.7008‐2A>C and a germline mutation at p.E2877* (p.Glu2877Ter), chr13:g.32945234G>T. The mutation at p.E2877* was observed in the ClinVar allele ID 67304 (Fig. 4b). The patient's son, who was diagnosed with breast cancer 2.5 years previously at 49 years of age, shared this germline mutation.

## 4 DISCUSSION

Prostate cancer is the most frequent cancer in men and the second leading cause of male cancer death in Western countries, and incidence is rising in Asia.^7^ The strongest risk factor for PCa is a family history, and germline *BRCA2* mutations confer the highest risk.^8^, ^9^ BRCA2 is critical for the repair of double strand breaks (DSBs) in genomic DNA by homologous recombination (HR). In response to DSBs, BRCA2 localizes RAD51 to the damaged site and mediates repair. Castration‐resistant PCa is frequently associated with genomic aberrations of DDR‐related genes, including BRCA2.^3^, ^10^, ^11^, ^12^

Prostate cancer associated with *BRCA2* mutation may be particularly responsive to platinum‐based chemotherapy. Platinum agents induce intra‐strand and inter‐strand cross‐linked DNA damage, which requires HR for repair. Loss of BRCA2 function in ovarian and endometrial cancer was associated with increased sensitivity to cisplatin, so patients with DDR alterations are thought to be sensitive to platinum agents. Indeed, our case patient showed a strong response to platinum‐based chemotherapy. Brain metastasis from PCa is rare and usually associated with particularly aggressive forms with widespread metastases which has poor overall survival (3−6 months).^13^, ^14^ Resection and subsequent radiation therapy has been reported to reduce recurrence risk of brain metastasis from PCa. The brain and lung metastatic lesions in this patient responded to platinum‐based chemotherapy. For further treatment, radiation therapy can be selected after acquired resistance to platinum‐based chemotherapy.^15^ Moreover, some of these DDR‐related genes are actionable targets by PARP inhibitors.^16^, ^17^

Prostate cancers with neuroendocrine differentiation include adenocarcinoma of NEPC, well‐differentiated neuroendocrine tumor, small‐cell neuroendocrine carcinoma, and large‐cell neuroendocrine carcinoma.^4^ *De novo* NEPC is a rare and highly aggressive malignant cancer.^2^, ^14^, ^18^ Under selection pressure by modern AR‐directed therapy, AR‐positive adenocarcinoma can dedifferentiate into a small‐cell neuroendocrine‐like tumor or treatment‐emergent neuroendocrine PCa (t‐NEPC). Recently, Beltran *et al*. reported that DDP gene aberrations were rare in t‐NEPC. On the other hand, frequent biallelic alterations in DDR genes were identified in a minority of patients with *de novo* NEPC,^2^ suggesting sensitivity to platinum agents or PARP inhibitors.

Advances in germline genetics and targeted therapeutics have created new treatment opportunities for aggressive PCa.^19^ However, the choice of optimal treatment is still a challenge as there are no consensus guidelines on treatment for cases with specific mutations. In the United States, the Prostate Cancer Clinical Trials Consortium was established in 2017 to address the growing problem of translating genetic information into therapeutics.^20^ The consortium assessed the current status of genetic testing practices in men with PCa and suggested greater emphasis on genetic testing results for treatment decisions, testing of family members, and further research for establishing associations among germline DDR gene mutations, PCa phenotypes, and treatment responses. In our case, the patient's son had a past history of breast cancer and both patients desired genomic sequencing analysis, which revealed a shared *BRCA2* mutation at p.E2877*. To our knowledge, this is the first case report in a Japanese patient with PCa exhibiting NEPC somatic and germline *BRCA2* mutations who responded to platinum‐based chemotherapy.

The analysis reports were discussed and reviewed at a genome expert conference consisting of medical oncologists, molecular oncologists, pathologists, medical geneticists, clinical laboratory technicians, bioinformaticians, genetic counselors, pharmacists, and nurses. The final report including information regarding the recommended treatment based on genomic profiling was confirmed after approval at this conference, after which the report was disseminated to the physicians and patient.

We present the first Japanese case of an 81‐year‐old male with metastatic adenocarcinoma of NEPC accompanied by brain metastasis and a *BRCA2* mutation. He had a 51‐year‐old son with breast cancer history sharing the same mutation, implying that *BRCA2* mutations are associated with elevated cancer risk among Japanese males.

## DISCLOSURE STATEMENT

None declared.

## AUTHOR CONTRIBUTIONS

Conception and design of the study: TK, HN and MO. Acquisition and analysis of data: TK, HH, EA, KM, TH and HN. Drafting the manuscript and figures: TK, SM, HN and MO.
